# A case of IgG4-related interstitial nephritis with ureteral obstruction: case report and literature review

**DOI:** 10.1186/s12894-023-01253-2

**Published:** 2023-04-28

**Authors:** Xiao-qing Cai, Zhi-bin Chen, Huai-huai Chen, Yan-qiu Zheng, Xu-guang Yu

**Affiliations:** Department of Nephrology, People’s Hospital of Yueqing, Yueqing Hospital Affiliated to Wenzhou Medical University, Yueqing, 325600 Zhejiang China

**Keywords:** IgG4-RD,ureter mass, Hydronephrosis, Hormone therapy

## Abstract

**Background:**

IgG4-related disease (IgG4-RD) is a newly discovered systemic disease that can affect any organ or tissue in the body. IgG4-related kidney disease (IgG4-RKD) is relatively rare but essential to IgG4-RD. However, there are few reports of IgG4-RD mimicking malignant ureteral tumors leading to hydronephrosis. We report here a rare case of IgG4-RD involving the ureter.

**Case presentation:**

An 87-year-old man presented to our nephrology department with anorexia, nausea, and acute kidney injury in November 2020. Urinary computed tomography (CT) examination revealed a right lower ureter mass with right renal and ureter hydronephrosis. The serum level of IgG4 was 1890 mg/dL, and the concurrently renal biopsy revealed extensive infiltration of IgG4-positive plasma cells in renal interstitium, which was diagnosed as IgG4-associated tubule-interstitial nephritis(IgG4-TIN). The renal function improved significantly after double-J tube implantation of the right ureter and moderate-dose hormone therapy. The serum IgG4 decreased to the normal range, and the right lower ureter mass almost disappeared after one year of low-dose hormone maintenance therapy.

**Conclusion:**

IgG4-RD can present as a mass in the renal pelvis and (or) ureter, leading to hydronephrosis. Therefore, early recognition of this disease is significant. Most patients respond well to hormonal therapy to avoid surgical treatment due to misdiagnosis as malignant tumors, causing secondary harm to patients.

## Background

IgG4-RD is a newly discovered systemic disease in the past 20 years, which can affect any organ or tissue in the body [1]. IgG4-RKD is relatively rare but an essential component of IgG4-RD. The most common renal manifestations of IgG4-RKD are tubulointerstitial nephritis and glomerular lesions [2]. However, there are few reports of IgG4-RD mimicking malignant ureteral tumors leading to hydronephrosis. We report here a rare case of IgG4-RD involving the ureter.

## Case presentation

**Fig. 1:**
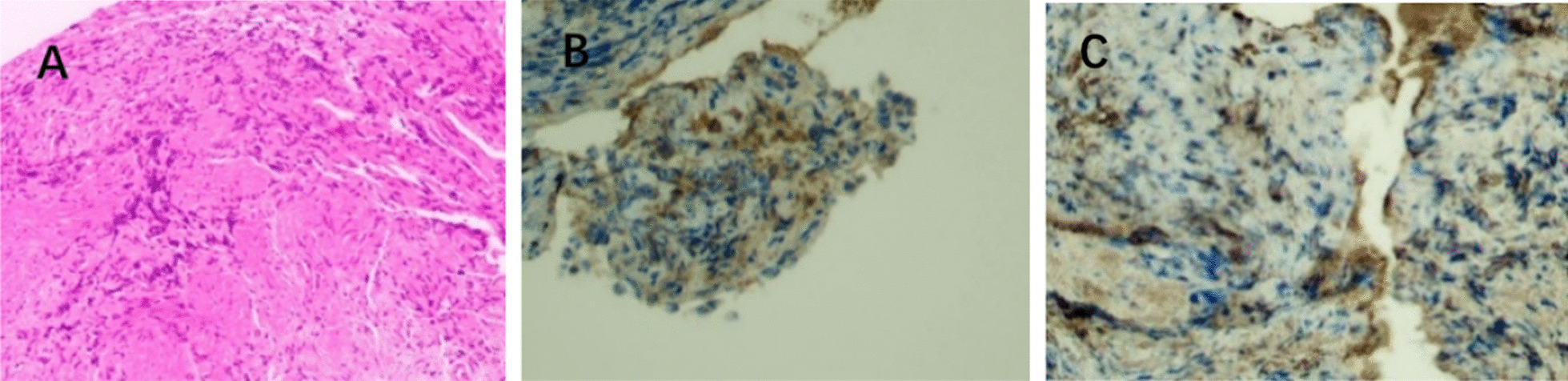
**A** HE staining. Extruded and deformed fibrous tissue of ureteral tissue (× 100). **B** Immunohistochemical staining. A small amount of IgG-positive plasma cells in the ureter (× 200). **C** Immunohistochemical staining. A small amount of IgG4-positive plasma cells in the ureter (× 200)

An 87-year-old male presented with a history of hypertension for over 50 years. He had type 2 diabetes for more than ten years, coronary heart disease for more than ten years, and "right lobectomy" for "right lung cancer" 16 years ago. The patient had a history of chronic renal insufficiency with basal serum creatinine around 187 μmol/L. In November 2020, he came to our outpatient department due to anorexia and nausea for half a month. His biochemical test results suggested that serum creatinine(Scr) was 428 μmol/L, urea nitrogen(BUN) was 20.9 mmol/L, the complement C3 was 0.53 g/L, C4 was standard, the IgG was 25.65 g/L, the blood eosinophil was 0.73 × 109/L, the total IgE was 453.3 IU/ mL, and the 24-h urinary protein quantification was 2.38 g. Urinary CT examination revealed a mass in the right lower ureter with the right renal and ureter hydronephrosis. Ultrasonography showed generalized superficial lymph node enlargement throughout the body. Due to the patient's previous history of lung cancer resection, the right ureteral mass was suspected to be a ureteral malignant tumor. We performed a urine examination three times to find abnormal cells, which were all negative. Cystoscopy and right ureteroscopy were performed by the urology department, which showed local bulging of the right ureteral orifice, ranging from about 2.5 × 1.0 cm, and stenosis of the right ureteral orifice, and an F6 double-J tube was indwelled in the lower segment of the right ureter. A biopsy was taken on a right ureteral orifice bulge, and a histopathological examination revealed the extruded and deformed fibrous tissue (Fig. 1A). Immunohistochemistry showed small IgG-positive and IgG4-positive plasma cells (Fig. 1B and C).

**Fig. 2:**
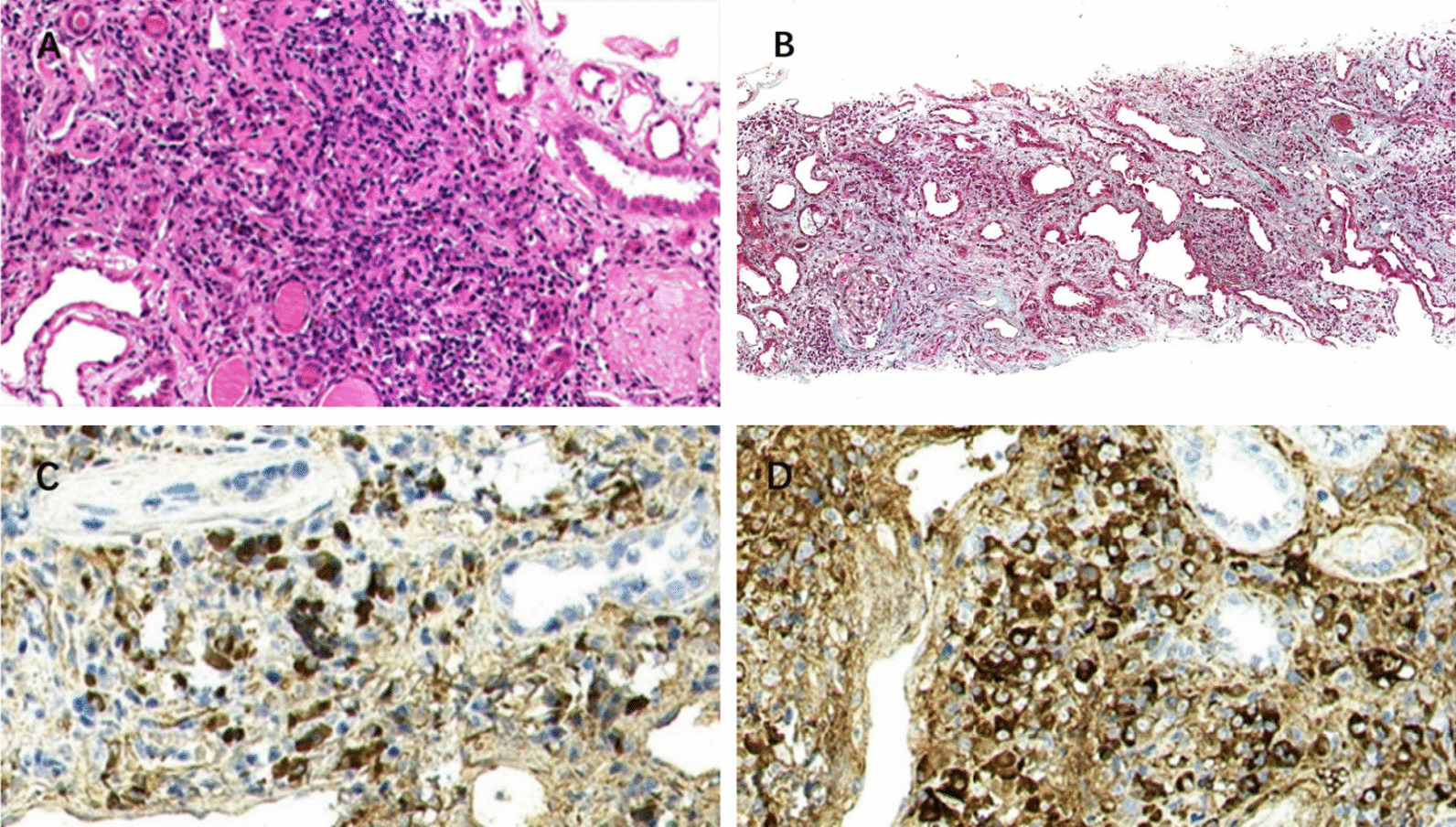
**A** HE staining. Numerous plasma cells are seen in the renal interstitium (× 200). **B** Masson staining. Renal interstitial mat striatum fibrosis (× 100). **C** Immunohistochemical staining. IgG4 renal interstitial focal cells were positive, with Several fields more significant than 25/HPF (× 400). **D** Immunohistochemical staining. IgG is positive for renal interstitial focal cells, IgG4/IgG = 35% (× 400)

Traced serum IgG4 1890 mg/dL was significantly increased, and IgG4-RD was speculated. Renal biopsy revealed proliferation of mesangial cells and matrix proliferation, diffuse homogeneous thickening of the basement membrane, and diffuse fusion of foot processes. Abundant IgG4-positive plasma cells were seen in the renal interstitial(Fig. 2A), and several fields were more significant than 25 lenses per high power, the IgG4 + : IgG ratio is = 35%(Fig. 2C and D), and visible storiform pattern fibrosis (Fig. 2B). The diagnosis was consistent with diabetic nephropathy with IgG4-TIN. Therefore, it was considered that the patient's ureteral mass was IgG4-RD.

**Fig. 3:**
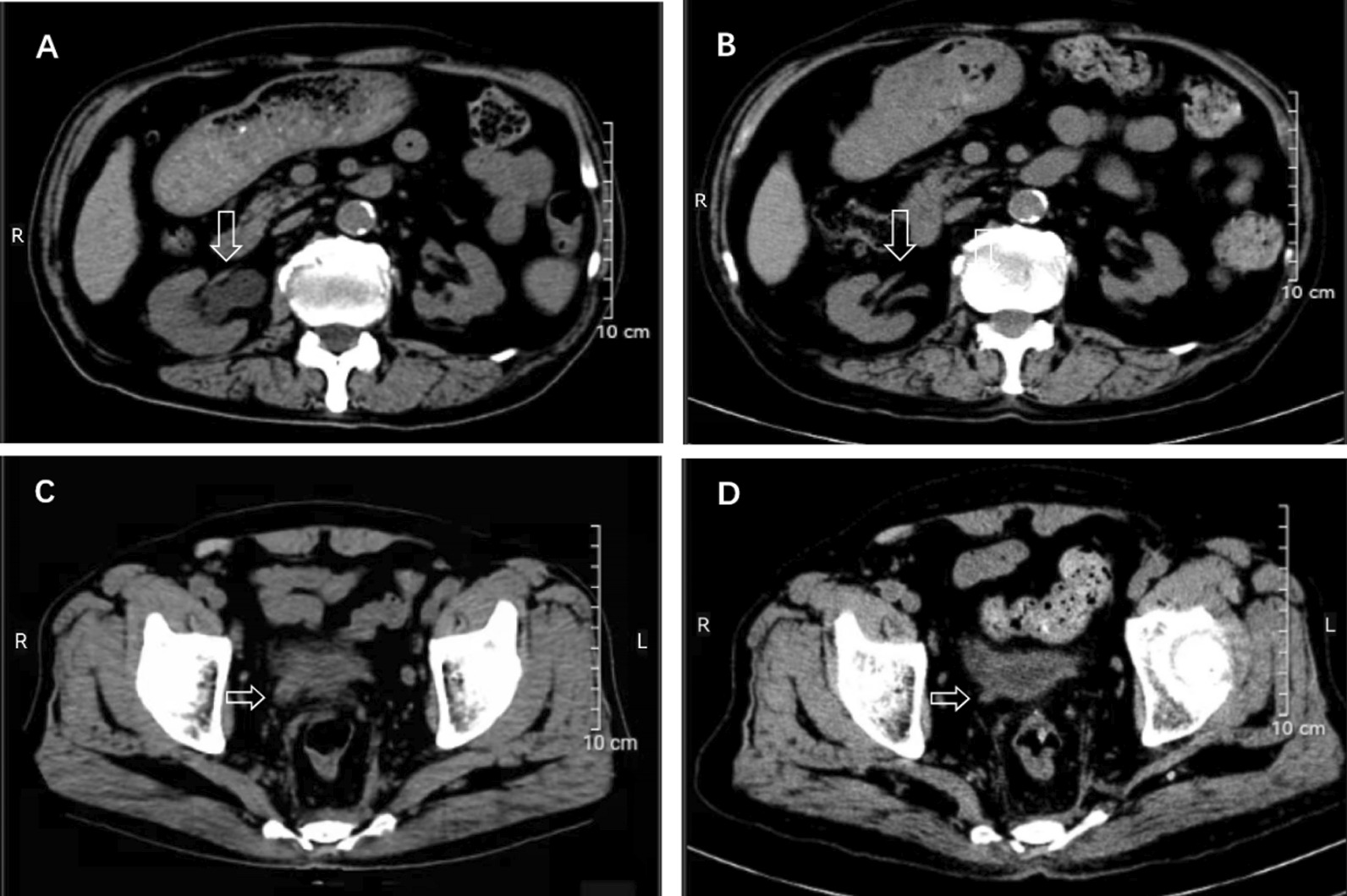
**A** CT image of the urinary system in December 2020, showing the right renal and ureter hydronephrosis. **B** CT image of the urinary system in December 2021 showed that the right renal and ureter hydronephrosis had disappeared. **C** CT image of the urinary system in December 2020 showed patchy soft tissue-like density shadows at the bladder inlet at the lower end of the right ureter. **D** CT image of the urinary system in December 2021 showed that patchy soft tissue-like density shadows at the bladder inlet had disappeared

After implantation of the double-J tube of the right ureter, the patient's serum creatinine decreased to 279 μmol/L. Half a month later, treated with 32 mg/ day methylprednisolone tablets, and we reduced to 16 mg/ day five weeks later. After two months of treatment, the serum creatinine was 196 μmol/L and IgG4 171 mg/dL. Then decrease methylprednisolone to one tablet every 12 weeks. The patient has been followed up for one year, the dose of methylprednisolone tablets has been reduced to 4 mg/day, the serum creatinine is stable at around 200 μmol/L, and the IgG4 is 59 mg/dL. On reexamination, the systemic, superficial lymph nodes had been significantly reduced, the right ureteral mass and hydrops had disappeared (Fig. 3), and removed the double-J tube after one year. Therefore, we considered that the patient's underlying chronic kidney disease was diabetic nephropathy, and the main factors of acute exacerbation of renal insufficiency were IgG4-related ureteral obstruction and IgG4-TIN.

According to the 2019 American College of Rheumatology (ACR) and European League Against Rheumatism (EULAR) classification criteria for IgG4-related disease [3], this case was scored as follows: Histopathology: Dense lymphocytic infiltration(+ 4); Kidney immunostaining: the IgG4 + : IgG ratio is < 40%, but IgG4 + cells/hpf is > 10(+ 7); Serum IgG4 concentration: > 5 × upper limit of normal(+ 11); Hypocomplementemia(+ 6). Total points are 28 > 20, and the diagnosis of IgG4-RD is precise. Combined with his ureteral mass, which was fibrous tissue that was slightly positive for IgG and IgG4, and the sound effect of hormone therapy, we comprehensively considered that this patient was a rare case of IgG4-RD involving both renal interstitium and ureter.

## Discussion and conclusions

In 2001, Hamano et al. found that patients with Autoimmune pancreatitis (AIP) had elevated serum IgG4 concentrations and massive IgG4-positive plasma cell infiltration in the diseased tissues [1]. It was then reported in other organs as a systemic disease. Common anatomic sites affected were the pancreas, salivary gland, lung, periorbital tissues, liver, breast, prostate, thyroid, and kidney. Histologically, it is characterized by an inflammatory infiltrate consisting mainly of lymphocytes and IgG4-positive plasma cells, without cytological atypia, and storiform pattern fibrosis [4–6]. In 2007, Zen et al. named such diseases as IgG4-related disease (IgG4-RD) [7], which has been widely recognized internationally. IgG4-RKD is most commonly founded in renal tubulointerstitial nephritis and glomerular diseases, especially membranous nephropathy [2]. However, only a few reports of IgG4-RD appear in the renal pelvis and(or) ureter. However, in this article, we report an unusual case of IgG4-RD.

Combined with this patient, the authors of this paper searched for reports worldwide in the past 20 years on IgG4-RD involving the renal pelvis and(or) ureter (Table 1) [1, 4, 8–35]. We found that most were case reports, with most cases reported by Japanese and Chinese urologists. Adding to this case, a total of 36 cases were summarized in the table, of which 26 cases (72.1%) were male, mostly unilateral renal pelvis and(or) ureter involvement(91.7%). Twenty-two patients (61.1%) showed hydronephrosis. Most IgG4-RD involving the ureter showed retroperitoneal fibrosis encasing the ureter, or inflammatory pseudotumor of the ureter, while thickening of the ureteral wall occurs and is easily confused with ureteral malignancies. Ureteral carcinoma was considered in 26 cases (72.2%), but IgG4-RD was pathologically diagnosed after nephroureterectomy or partial ureterectomy. However, only seven patients (19.4%) improved after hormone therapy alone and were not treated surgically. This patient was fortunate; after our discussion and literature search, we highly suspected that the patient's ureteral lesion was IgG4-related, significantly improving after relieving ureteral obstruction and hormone therapy, avoiding the trauma of surgery for the patient.

**Table 1:** Summary of IgG4-related disease in the renal pelvis and(or) ureter

Case	Author	Age(y)	Sex	Side	IgG4 (mg/dL)	Hydronephrosis	Extra-renal lesions	Operation	Diagnosis	Steroid
1	Hamano et al	60	M	Lt	265	+	AIP	Nephroureterectomy	RPF	40 mg
2	Hamano et al	74	M	Rt	965	+	AIP	Partial ureteral resection	RPF	Yes
3	Hamano et al	63	M	Lt	1540	+	AIP	–	RPF	–
4	Buyl et al	65	M	Lt	–	+	Nk	Nephroureterectomy	RPF	–
5	Kamisawa et al	75	M	Lt	240	+	AIP	Partial ureteral resection	RPF	30 mg
6	Harper et al	13	M	Lt	–	+	–	Politano-Leadbetter	IPT	–
7	Montgomery et al	70	M	Rt	–	+	Nk	Nephroureterectomy	IMT	–
8	Hosokawa et al	66	M	Lt	–	+	Nk	Nk	IPT	–
9	Hattori et al	60	F	Lt	Nk	–	–	Nephroureterectomy	IPT	Nk
10	Joo et al	53	M	Rt	–	+	Nk	Nephroureterectomy	ISU	–
11	Kojima et al	47	F	Bi	478	Nk	Nk	–	PCCD	Yes
12	Kojima et al	73	M	Lt	412	Nk	Nk	Nephroureterectomy	PCCD	–
13	Abe et al	39	M	Lt	233	+	–	Partial ureteral resection	RPF	–
14	Kim et al	45	M	Lt	–	+	–	Nephroureterectomy	IPT	–
15	Kim et al	47	M	Lt	–	–	–	Partial ureteral resection	IPT	–
16	Kim et al	84	F	Rt	–	+	urothelial carcinoma	Nephroureterectomy	IPT	–
17	Uehara et al	58	M	Lt	462	+	IgG4-MD	left nephrectomy	RPF	30 mg
18	Takata et al	80	M	Rt	220	+	–	nephroureterectomy	IgG4-RD	–
19	Takata et al	68	M	Rt	202	–	pituitary gland tumor	–	IgG4-RD	Yes
20	Yoshino et al	71	M	Lt	847	+	Retroperitoneal mass	–	RPF	20 mg
21	Nomura et al	79	F	Lt	206	+	IgG4-TIN	nephroureterectomy	ISU	Yes
22	Marand et al	77	M	Lt	–	–	–	cystectomy	IPT	–
23	Marand et al	82	F	Lt	–	+	–	nephroureterectomy	IPT	Yes
24	Inoue et al	49	F	Bi	469	–	–	–	RPF	40 mg
25	Tsusaka et al	69	M	Lt	874	–	pulmonary hilar mass	nephroureterectomy	IPT	30 mg
26	Wang YW et al	54	F	Lt	1860	+	lymphadenectasis	nephroureterectomy	IPT	Yes
27	CAI Y et al	80	F	Lt	–	–	–	nephroureterectomy	IPT	–
28	Ramasamy et al	37	M	Rt	2530	–	–	–	IPT	–
29	Park HG et al	75	M	Rt	–	+	–	nephroureterectomy	IPT	–
30	Zhang H et al	53	F	Rt	3250	–	–	nephroureterectomy	IPT	–
31	Lei WH et al	70	M	Lt	913	+	–	nephroureterectomy	RPF	60 mg
32	Zhong W et al	64	M	Rt	–	+	Nk	nephroureterectomy	IgG4-RD	–
33	Yamamoto et al	69	M	Rt	360	–	renal cell carcinoma	–	RPF	30 mg
34	Sangsoada et al	75	F	–	–	–	AIP	–	IPT	Yes
35	Tomoyuki et al	72	M	Lt	384	–	–	Partial ureteral resection	RPF	–
36	Our case	87	M	Rt	1890	+	IgG4-TIN	–	IgG4-RD	32 mg

According to consensus statement on the pathology of IgG4-related disease [36]

*F* female, *M* male, *Nk* not know, *Lt* left, *Rt* right, *Bi* bilateral, *IMT* inflammatory myofibroblastic tumor, *RPF* retroperitoneal periureteral fibrosis, *IPT* inflammatory pseudotumor, *ISU* idiopathic segmental ureteritis, *PCCD* plasma cell type of Castleman's disease, *MD* Mikulicz’s disease

According to the reports of Ramasamy V et al. [28] and Lei WH et al. [31], IgG4-RD has the possibility of spontaneous remission, manifested as automatic reduction or even regression of ureteral mass. Still, the overall spontaneous remission rate is relatively low. Due to the short follow-up time in most cases, whether there was a recurrence in the late stage of treatment was not mentioned. However, according to the prospective cohort study conducted by Peking Union Medical College Hospital in China between 2011 and 2019, a total of 65 cases were collected IgG4-RD, 14 patients (21.5%) were found to have renal pelvis or ureter involvement, while 16 patients (24.6%) had a re-elevation of serum IgG4 during follow-up. Four patients (6.2%) experienced clinical relapse, and all presented with worsening renal impairment [37]. This patient has been followed up for one year without signs of recurrence. In the case reported by Kim et al. [18], the patient was found to have a ureteral mass considered as IgG4-RD on one side, while ureteral epithelial lymphoma in the reported case by Uehara et al. [19]. The authors stated that MALT lymphoma might be related to IgG4-RD. In addition, Yamamoto et al. [38] reported in 2012 that among 106 patients with IgG4-RD, 11 patients developed malignant tumors during the follow-up period, with the incidence of malignant tumors of 10.4%, significantly higher than that of ordinary people. Therefore, it is recommended that clinicians follow up on all patients diagnosed with IgG4-related diseases for a long time and closely track the possibility of recurrence and the possibility of malignancy.

In conclusion, IgG4-RD can present as a mass in the renal pelvis and (or) ureter, leading to hydronephrosis and resembling a ureteral tumor. Although IgG4-RD may have many clinical manifestations with multiple organ involvement, but there are few reports on such participation of the ureter. Therefore, early recognition of this disease is significant. Most patients respond well to hormonal therapy to avoid surgical treatment due to misdiagnosis as malignant tumors, causing secondary harm to patients.

## Data Availability

All data generated or analysed during this study are included in this published article (and its supplementary information files).

## Abbreviations

IgG4-RD: IgG4-related disease
IgG4-RKD: IgG4-related kidney disease
IgG4-TIN: IgG4-associated tubule-interstitial nephritis
Scr: Serum creatinine
BUN: Urea nitrogen
ACR: American College of Rheumatology
hpf: High power field
CT: Computed tomography
EULAR: European League Against Rheumatism
AIP: Autoimmune pancreatitis
